# Knowledge, Attitude, and Practice (KAP) Regarding the Use of Artificial Intelligence in Hospital Settings in Mardan, Khyber Pakhtunkhwa, Pakistan

**DOI:** 10.7759/cureus.75355

**Published:** 2024-12-09

**Authors:** Faizan Sadiq, Faran Sadiq, Ruba Gul, Fatima Zuhra, Muhammad Khalid Khan, S Mohsin Ali Shah, Faryal Uzma, Noor Ul Haram Khattak, Waqas Alam, Misbah Ullah Khan

**Affiliations:** 1 Pediatrics Department, Mardan Medical Complex, Mardan, PAK; 2 Accident and Emergency, Lady Reading Hospital, Peshawar, PAK; 3 Pediatrics Department, Khyber Teaching Hospital, Peshawar, PAK; 4 Anesthesia Department, Lady Reading Hospital, Peshawar, PAK

**Keywords:** artificial intelligence (ai), doctors, hospital setting, knowledge attitude and practice, qualitative research

## Abstract

Background

Artificial intelligence (AI) is revolutionizing healthcare globally by enhancing diagnostic accuracy, predicting patient outcomes, and enabling personalized treatment plans. However, in low- and middle-income countries (LMICs) like Pakistan, the integration of AI into healthcare is limited due to challenges such as lack of funding, provider resistance, and inadequate training. Despite these barriers, there is growing interest among healthcare providers in understanding and adopting AI technologies to improve professional efficiency.

Objective

To determine knowledge, attitude, and practice (KAP) regarding the use of artificial intelligence (AI) among doctors in a hospital setting.

Method

This cross-sectional study was conducted at Mardan Medical Complex, Mardan, from January 2023 to March 2023. A total of 150 doctors from various departments participated by completing a validated questionnaire designed to assess their KAP regarding AI. The questionnaire, consisting of nine close-ended questions, was specifically designed and distributed to participants through social media applications, including WhatsApp (California, USA) and email, to maximize accessibility. It included structured questions rated on Likert scales to quantify the levels of knowledge, attitude, and practice. Participants were also asked about their exposure to AI-related training or professional work. Descriptive analysis was performed to determine the frequency and percentage of responses.

Results

The mean ± SD of age in this study was 36.95 ± 8.58 years. Male participants were 72.66%, while 27.33% were females. The response to the questions showed that the majority of participants (66%) knew the term AI; however, they were unsure regarding its use in healthcare. The majority of doctors (67.33%) had positive thoughts about the possibility of using AI in health management. Importantly, the majority of participants (72.66%) had never had a chance to do any AI-related work in their professional lives. The assessment of KAP showed that the majority of doctors had a medium level of knowledge (36.66%), a high level of attitude (57.55%), and a low level of practice (65.66%) regarding AI.

Conclusions

These results conclude that the knowledge regarding AI's use in healthcare is medium and its use in clinical practice is low; however, doctors have a high level of interest in applying AI to improve their professional efficiency.

## Introduction

The medical and healthcare industries are on the brink of a revolutionary shift, with artificial intelligence (AI) offering immense potential for future healthcare initiatives. AI has a wide array of applications, such as predicting patient outcomes, enhancing diagnostic accuracy, and developing personalized treatment plans [1]. Furthermore, AI is profoundly impacting the way future healthcare professionals and medical students interact within the healthcare ecosystem, creating new opportunities for learning and professional development [2].

High-income countries have invested substantial budgets in implementing AI within their healthcare sectors, demonstrating positive acceptance, especially in medical education. A 2020 study conducted among medical students in the United Kingdom found that 88% of participants recognized AI’s significant role in the future of healthcare. However, only 9.2% reported having received any formal education related to AI [3,4].

In contrast, low- and middle-income countries (LMICs) like Pakistan face a significant shortage of information, funding, and research on the integration of AI in healthcare. Despite the limited exposure, studies conducted in Pakistan indicate that medical students are highly interested in learning about AI [5]. This interest is particularly noteworthy given AI’s potential to transform healthcare delivery, even in resource-constrained settings [4].

While AI algorithms are sometimes used in radiology, their application in other medical specialties, such as oncology, psychiatry, dermatology, ophthalmology, cardiology, pathology, and neuroscience, remains limited. AI technologies have been utilized globally for various applications, including diagnostic imaging, predictive analytics, and patient management tools, highlighting their potential to transform healthcare practice [6,7]. AI education initiatives are being introduced for postgraduate trainees in various hospital departments to supplement undergraduate learning [8]. One significant step in this direction was an AI program initiated by the president of Pakistan. However, the application of AI in healthcare has encountered challenges, including provider resistance, budget limitations, a shortage of skilled medical professionals, and difficulties establishing diagnostic protocols essential for algorithm development. Additional barriers include public perception, fears of job displacement, societal and cultural obstacles, confidentiality concerns, and medico-legal issues [9].

AI plays a transformative role in healthcare by facilitating accurate predictions, which can aid providers in decision-making and treatment planning. The implementation of AI-based approaches has the potential to improve precision in multiple medical fields. A review conducted by Khan MI highlighted the use of AI in anesthesiology, cardiology, pulmonology, radiology, pathology, and other critical areas. The study found that while progress has been made, AI adoption in Pakistan’s healthcare sector is still limited. Researchers emphasized that Pakistan has yet to fully leverage the benefits of this advanced technology, indicating that the sector remains distant from fully harnessing AI's potential [10].

Given this situation, it is clear that there is significant untapped potential for AI in Pakistan's healthcare system. To effectively utilize AI, a comprehensive understanding of the current baseline is essential. This study, therefore, aims to evaluate the knowledge, attitude, and practice (KAP) of doctors regarding AI usage in hospital settings at Mardan Medical Complex, Mardan, Khyber Pakhtunkhwa. This first-of-its-kind study in Pakistan, focusing exclusively on doctors within a healthcare institution, will address a notable gap in the literature and provide valuable insights from healthcare providers at the grassroots level.

## Materials and methods

Study design and setting

This was a cross-sectional descriptive study conducted at Mardan Medical Complex, Mardan, Khyber Pakhtunkhwa, Pakistan. The study spanned three months, from January 1, 2023, to March 31, 2023. The hospital is a tertiary care center that caters to a diverse patient population and employs healthcare providers from multiple specialties, providing a representative sample of practicing physicians for this study.

Study population and sampling

The study population included doctors working across various departments of the hospital, irrespective of their designations or years of experience. The sample size was 150, calculated using a precision level of 8%, a prevalence rate of 63.5% for AI knowledge among healthcare providers (based on a previous study [11]), a 95% confidence interval, and an expected 10% dropout rate. Convenience sampling was employed due to logistical constraints and time limitations. Several constraints were noted in this study, including potential selection bias due to voluntary participation and the reliance on social media for survey distribution, which may not represent the entire healthcare provider population. Additionally, self-reported data could introduce response bias.

Inclusion and exclusion criteria

All doctors currently employed at Mardan Medical Complex and consenting to participate were included in the study. Those who provided incomplete responses to the questionnaire or did not consent were excluded.

Questionnaire design and validation

A structured questionnaire, adapted from validated tools used in previous studies on similar topics [11-13], was used to assess knowledge, attitudes, and practices (KAP) regarding AI. The questionnaire consisted of nine close-ended questions, divided equally across three domains: knowledge (three questions), attitude (three questions), and practice (three questions). Each question had three possible responses, with responses categorized as follows: Option A: High/good level, Option B: Medium level, Option C: Low/poor level.

To ensure validity and reliability, the questionnaire was pretested on a small subset of doctors (n=10) not included in the final analysis. Feedback from the pre-test was used to refine question phrasing and ensure clarity. Internal consistency was assessed using Cronbach’s alpha, yielding an acceptable value of 0.78.

Data collection procedure

Participants were recruited via direct communication and departmental announcements. After obtaining informed consent, the questionnaire was distributed electronically through social media platforms, including WhatsApp and email, to enhance accessibility and response rates. Reminders were sent to non-respondents one week after the initial distribution. The study's objectives were explained in detail, and confidentiality was assured.

Ethical considerations

The study was approved by the Institutional Ethical Review Board of Mardan Medical Complex (391/BKMC). Participation was voluntary, and informed consent was obtained electronically before questionnaire distribution. Data confidentiality was maintained by anonymizing responses during data analysis.

Data analysis

Data were entered into IBM Corp. Released 2017. IBM SPSS Statistics for Windows, Version 25.0. Armonk, NY: IBM Corp. for statistical analysis. Quantitative variables such as age were expressed as mean ± standard deviation (SD), while qualitative variables such as gender, knowledge, attitude, and practice levels were expressed as frequencies and percentages. Descriptive statistics were used to analyze the responses for each domain of the KAP questionnaire. Composite scores for each domain were calculated by summing responses to the respective questions, allowing for an overall assessment of KAP.

## Results

The mean age of the study participants was 36.95 ± 8.58 years, with an age range spanning from 25 to 57 years. Among the participants, 72.66% were male, while 27.33% were female. The demographic details are presented in Table 1.

**Table 1:** Demographics of study participants (n=150)

Demographics		
Age (Mean±SD) years		36.95±8.58
Gender	Male n (%)	109 (72.66)
	Female n (%)	41 (27.33)
Department	Medicine n (%)	21 (14)
	Surgery n (%)	19 (12.66)
	Gynaecology n (%)	17 (11.33)
	Pediatrics n (%)	16 (10.66)
	Ophthalmology n (%)	8 (5.33)
	ENT n (%)	7 (4.66)
	Cardiology n (%)	7 (4.66)
	Dermatology n (%)	6 (4)
	Orthopedics n (%)	6 (4)
	Outpatients Dept. n (%)	5 (3.33)
	Radiology n (%)	5 (3.33)
	Oncology n (%)	5 (3.33)
	Pulmonology n (%)	5 (3.33)
	Psychiatry n (%)	4 (2.66)
	Pathology n (%)	4 (2.66)
	Gastroenterology n (%)	4 (2.66)
	ICU n (%)	3 (2)
	Neurosurgery n (%)	3 (2)
	Emergency n (%)	3 (2)
	Administration n (%)	2 (1.33)
Designation	Professor n (%)	4 (2.66)
	Assoc. Prof. n (%)	7 (4.66)
	Asst. Prof. n (%)	11 (7.33)
	Sen. Registrar n (%)	17 (11.33)
	Post Graduate Trainee n (%)	61 (40.66)
	Medical Officer n (%)	44 (29.33)
	Sen. Medical officer n (%)	4 (2.66)
	Deputy Med. Superintendent n (%)	2 (1.33)

Responses to the survey questions indicated that the majority of participants (70%) were familiar with the term AI but were uncertain about its application in healthcare. Additionally, most doctors expressed a positive outlook regarding the potential use of AI in health management (67.33%). It is noteworthy that a significant portion (72.66%) of participants had never engaged in any AI-related tasks within their professional careers. Detailed responses are provided in Table 2.

**Table 2:** Response to the questions regarding KAP on AI (n=150)

Sequence	Questions	Options	Findings n (%)
Knowledge			
1	Do you know about AI	A n (%)	70 (46.66)
		B n (%)	65 (43.33)
		C n (%)	15 (10)
2	Artificial Intelligence (AI) has any association with healthcare	A n (%)	50 (33.33)
		B n (%)	65 (43.33)
		C n (%)	35 (23.33)
3	Can you list at least two applications of AI in medicine?	A n (%)	29 (19.33)
		B n (%)	35 (23.33)
		C n (%)	86 (65.33)
Attitude			
1	How do you feel about the use of AI in healthcare?	A n (%)	101 (67.33)
		B n (%)	24 (16)
		C n (%)	25 (16.6)
2	Do you believe AI will significantly impact the future of medicine?	A n (%)	81 (54)
		B n (%)	35 (23.33)
		C n (%)	34 (22.66)
3	Would you be willing to undergo training in AI technologies during your medical education	A n (%)	77 (51.33)
		B n (%)	45 (30)
		C n (%)	28 (18.66)
Practice			
1	Have you ever done any projects/ research related to use of AI in health-related tasks?	A n (%)	18 (12)
		B n (%)	23 (15.33)
		C n (%)	109 (72.66)
2	How much have you used AI-based healthcare tools or applications?	A n (%)	24 (16)
		B n (%)	29 (19.33)
		C n (%)	97 (64.66)
3	How often do you seek information or updates on AI in healthcare?	A n (%)	27 (18)
		B n (%)	34 (22.66)
		C n (%)	89 (59.33)

The KAP assessment revealed that most participants had a medium level of knowledge, a high level of positive attitude, and a low level of practical application regarding AI. These findings are summarized in Table 3.

**Table 3:** Findings of KAP (n=150)

Components of KAP	Categories	Findings
Knowledge	High n (%)	49.66 (33.11)
	Medium n (%)	55 (36.66)
	Low n (%)	45.33 (30.22)
Attitude	High n (%)	86.33 (57.55)
	Medium n (%)	34.66 (23.10)
	Low n (%)	29 (19.33)
Practice	High n (%)	23 (15.33)
	Medium n (%)	28.66 (19.1)
	Low n (%)	98.33 (65.66)

## Discussion

Various studies have evaluated the knowledge, attitude, and practice (KAP) regarding AI among healthcare providers. However, most of these studies also included medical students and paramedic staff, making it difficult to determine the KAP among doctors solely.

A cross-sectional study conducted by Kalaimani G in India assessed the KAP of AI among dentists, including both doctors and students. The study used a self-structured questionnaire distributed online and found that 63.5% of participants were familiar with AI. Furthermore, 60% supported incorporating AI into dental curricula. The authors noted that while doctors were aware of AI, their ability to apply AI tools and models in medical practice was limited [11].

In Jordan, Al-Qerem W et al. conducted a study involving medical students that revealed moderate levels of AI knowledge. Although some AI tools were utilized for specific tasks, they were not extensively integrated into medical practice or education. Barriers such as limited information, accessibility issues, time constraints, and gaps in the curriculum were identified as obstacles to broader integration [14]. A similar study conducted by Al Hadithy ZA in Oman reported comparable findings among medical students [15].

Swed S’s study in Syria included both doctors and medical students and found that 70% of participants had some knowledge of AI, but only 23.7% used it in their professional work. The study reported a positive attitude toward AI, with 87.4% of participants recognizing its importance in healthcare protocols. The researcher emphasized that while participants lacked comprehensive knowledge of AI and its relevance in healthcare, they showed strong support for its integration into medical practice [12].

Ahmed Z’s online survey involving 470 doctors and medical students in Pakistan showed that 74% of doctors and 88.8% of students were aware of AI basics. However, only 27.3% of doctors and 19.4% of medical students were familiar with its practical application. A high proportion (76.7%) supported adding AI to medical curricula, indicating a strong positive attitude. This study concluded that, while knowledge and practical use were limited, there was a general eagerness to learn and adopt AI [13]. A study by Habib MM in 2024 similarly noted that Pakistani healthcare professionals had a positive attitude (66.4%) despite lacking comprehensive knowledge (78.7%) of AI [16].

In our study, the mean ± SD age of participants was 36.95 ± 8.58 years, with an age range of 25 to 57 years. Out of the total study population, 72.66% were male and 27.33% were female. Doctors from all departments participated, providing a comprehensive overview of the hospital’s workforce, including various designations and age groups. The results showed that 66% of participants were familiar with the term AI but were uncertain about its use in healthcare. Additionally, 67.33% had a positive attitude toward using AI in health management, yet 72.66% reported never having used AI in their professional work. The KAP assessment revealed medium knowledge (36.66%), high attitude (57.55%), and low practice (65.66%) levels among participants. These findings align with those of other studies, offering valuable insights into the current state of AI KAP among healthcare providers in our institution [11-13,16].

Despite the promising attitude of medical professionals across these studies, a recent systematic review emphasized the importance of including relevant AI education in medical curricula to foster understanding and application [17]. Similarly, a review focusing on the situation in Pakistan highlighted that, with available data and technological advancements, AI holds great potential for improving healthcare. The review suggested that addressing specific healthcare challenges, developing reliable AI models, and prioritizing patient safety, privacy, and trust are crucial for fully leveraging AI’s capabilities [18].

The primary limitation of our study is the small sample size, which may limit the statistical power of our findings. Additionally, this study was conducted in one medium-sized institution in a medium-sized city, meaning the results may not be generalizable across other regions or larger healthcare settings. While our findings provide initial insights into the relatively underexplored topic of AI use in our healthcare system, we recommend further research with larger samples to confirm these results. We advise readers to interpret our findings with caution given these limitations.

## Conclusions

These results conclude that knowledge regarding AI is medium, and its use in clinical practice is low; however, doctors have a high level of interest in applying AI to improve their professional efficiency. This indicates a clear need for targeted training programs and initiatives to equip healthcare providers with the necessary skills to integrate AI into routine medical practice. Incorporating AI into medical training and creating an enabling environment for its use can address current gaps in knowledge and practice, ultimately enhancing patient care and healthcare delivery. This study highlights the importance of proactive steps to ensure that advancements in AI are accessible and beneficial to all levels of the healthcare system.
